# Hygienic Precepts

**Published:** 1897-06

**Authors:** 


### Hygienic Precepts.

The late distinguished surgeon, Frank H. Hamilton, M. D., is credited with the formulation of the following decalogue:

1. The best thing for the inside of a man is the outside of a horse.

2. Blessed is he who invented sleep; but thrice blessed the man who will invent a cure for thinking.

3. Light gives a bronzed or tan color to the skin; but where it uproots the lilly it plants the rose.

4. The lives of most men are in their own hands, and, as a rule, the just verdict after death would be—*Felo de se.*

5. Health must be earned—it can seldom be bought.

6. A change of air is less valuable than a change of scene. The air is changed every time the wind is changed.

7. Mould and decaying vegetables in a cellar weave shrouds for the upper chambers.

8. Dirt, debauchery, disease and death are successive links in the same chain.

9. Calisthenics may be very genteel, and romping very ungenteel, but one is the shadow, and the other the substance, of healthful exercises.

10. Girls need health as much—nay, more than boys. They can only obtain it as boys do, by running, tumbling—by all sorts of innocent vagrancy. At least once a day girls should have their halters taken off, the bars let down, and be turned loose like young colts.—*Gross Medical College Bulletin.*

